# 2,2-Dichloro-1-(2-phenyl-1,3-oxazolidin-3-yl)ethanone

**DOI:** 10.1107/S1600536810002461

**Published:** 2010-01-27

**Authors:** Fei Ye, Ying Fu, Shuang Zhao

**Affiliations:** a College of Science, Northeast Agricultural University, Harbin 150030, People’s Republic of China

## Abstract

In the title mol­ecule, C_11_H_11_Cl_2_NO_2_, the oxazolidine ring is in an envelope conformation with the O atom forming the flap; the other four essentially planar ring atoms (r.m.s. deviation = 0.012 Å) form a dihedral angle of 91.1 (3)° with the phenyl ring. In the crystal structure, mol­ecules are linked by weak inter­molecular C—H⋯O hydrogen bonds, forming one-dimensional chains.

## Related literature

For general background to substituted oxazolidines see: Agami *et al.* (2004 ▶); Guirado *et al.* (2003 ▶); Tararov *et al.* (2003 ▶). For the bioactivity of related compounds, see: Hatzios *et al.* (2004 ▶); Daniele *et al.* (2007 ▶). For details of the synthesis, see: Fu *et al.* (2009 ▶). 
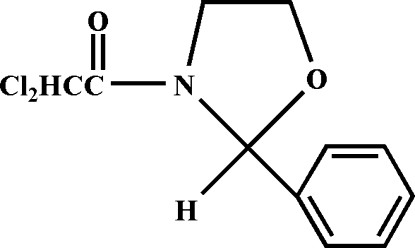


## Experimental

### 

#### Crystal data

C_11_H_11_Cl_2_NO_2_
*M* *_r_* = 260.11
Orthorhombic, 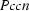

*a* = 19.1775 (13) Å
*b* = 10.6165 (7) Å
*c* = 11.3723 (8) Å
*V* = 2315.4 (3) Å^3^
*Z* = 8
Mo *K*α radiation
μ = 0.54 mm^−1^
*T* = 298 K
0.46 × 0.38 × 0.20 mm

#### Data collection

Bruker SMART CCD diffractometer
Absorption correction: multi-scan (*SADABS*; Sheldrick, 1996 ▶) *T*_min_ = 0.780, *T*_max_ = 0.897
16860 measured reflections
2846 independent reflections
2323 reflections with *I* > 2σ(*I*)
*R*_int_ = 0.022

#### Refinement

*R*[*F* ^2^ > 2σ(*F* ^2^)] = 0.040
*wR*(*F* ^2^) = 0.110
*S* = 1.04
2846 reflections
145 parameters
H-atom parameters constrained
Δρ_max_ = 0.41 e Å^−3^
Δρ_min_ = −0.29 e Å^−3^

### 

Data collection: *SMART* (Bruker, 1998 ▶); cell refinement: *SAINT* (Bruker, 1998 ▶); data reduction: *SAINT*; program(s) used to solve structure: *SHELXS97* (Sheldrick, 2008 ▶); program(s) used to refine structure: *SHELXL97* (Sheldrick, 2008 ▶); molecular graphics: *SHELXTL* (Sheldrick, 2008 ▶); software used to prepare material for publication: *SHELXTL*.

## Supplementary Material

Crystal structure: contains datablocks global, I. DOI: 10.1107/S1600536810002461/lh2978sup1.cif

Structure factors: contains datablocks I. DOI: 10.1107/S1600536810002461/lh2978Isup2.hkl

Additional supplementary materials:  crystallographic information; 3D view; checkCIF report

## Figures and Tables

**Table 1:** Hydrogen-bond geometry (Å, °)

*D*—H⋯*A*	*D*—H	H⋯*A*	*D*⋯*A*	*D*—H⋯*A*
C11—H11⋯O2^i^	0.98	2.40	3.312 (2)	156

Symmetry code: (i) 
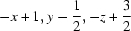
.

**Fig. 1.**
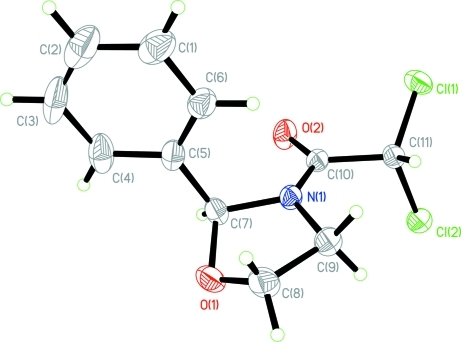
The molecular structure of the title compound, with the atom-labelling scheme.Displacement ellipsoids are drawn at the 30% probability level.

**Fig. 2.**
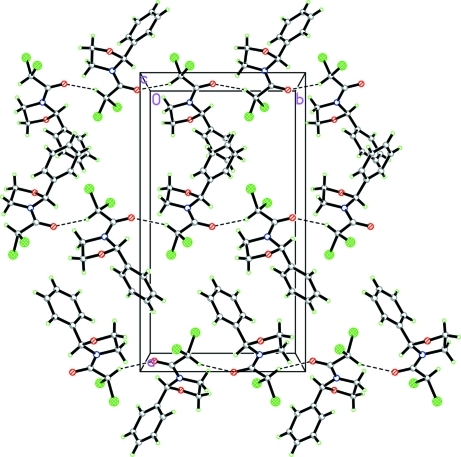
Part of the crystal structure of the title compound showing C-H···O hydrogen bonds as dashed lines.

## supplementary crystallographic information

### Comment

Substituted oxazolidines are important synthetic targets due to their biological activity (Agami *et al.*, 2004), pharmacological activity and their extensive use as chiral auxiliaries for the synthesis of many chiral compounds (Guirado *et al.*, 2003; Tararov *et al.* 2003). Dichloroacetemide compounds have been shown to act as herbicide safeners (Hatzios, 2004; Daniele *et al.*,  2007). As part of our ongoing investigations of oxazolidine derivatives we prepared the title compound and its crystal structure is reported herein.

The molecular structure of the title compound is shown in Fig.1. In the crystal structure, molecules are linked by weak intermolecular C—H···O hydrogen bonds to form one-dimensional chains (Fig. 2).

### Experimental

The title compound was prepared by a slightly modified literature procedure (Fu *et al.*, 2009).

Ethanolamine (4.1 g, 0.067 mol) and 7.1g (0.067mol) of benzaldehyde were mixed with 25mL of benzene. The reaction mixture was stirred for 1h at 306-308K. Then, the mixture was heated to reflux and water was evaporated, followed by cooling to 273K and 7.5 mL of 33% sodium hydroxide solution was added. 11.8 g (0.08mol) of dichloroacetyl chloride was added dropwise with stirring, keeping the temperature at 273-277K. Stirring was continued for 1.5h. The mixture was rinsed with water until the pH=7. The organic phase was dried over anhydrous magnesium sulfate and the benzene was removed under vacuum. The crude product was recrystallized with ethyl acetate and light petroleum, white crystals wre obtained. The yield was 58.2%. m.p. 374-377K.

The single-crystal suitable for X-ray structural analysis was obtained by slow evaporation of a solution of the title compound in petroleum ether and ethyl acetate at room temperature.

### Refinement

All H atoms were initially located in a difference Fourier map. The C—H atoms were then constrained to an ideal geometry, with C-H = 0.93 - 0.98Å and *U*_iso_(H) = 1.2*U*_eq_(C).

### Crystal data

**Table:**

C_11_H_11_Cl_2_NO_2_	*F*(000) = 1072.0
*M**_r_* = 260.11	*D*_x_ = 1.492 Mg m^−^^3^*D*_m_ = 1.492 Mg m^−^^3^*D*_m_ measured by not measured
Orthorhombic, *P**c**c**n*	Mo *K*α radiation, λ = 0.71073 Å
Hall symbol: -P 2ab 2ac	Cell parameters from 5877 reflections
*a* = 19.1775 (13) Å	θ = 2.8–27.9°
*b* = 10.6165 (7) Å	µ = 0.54 mm^−^^1^
*c* = 11.3723 (8) Å	*T* = 298 K
*V* = 2315.4 (3) Å^3^	Block, colourless
*Z* = 8	0.46 × 0.38 × 0.20 mm

### Data collection

**Table:**

Bruker SMART CCD diffractometer	2846 independent reflections
Radiation source: fine-focus sealed tube	2323 reflections with *I* > 2σ(*I*)
graphite	*R*_int_ = 0.022
φ and ω scans	θ_max_ = 28.3°, θ_min_ = 2.8°
Absorption correction: multi-scan (*SADABS*; Sheldrick, 1996)	*h* = −25→25
*T*_min_ = 0.780, *T*_max_ = 0.897	*k* = −14→14
16860 measured reflections	*l* = −15→15

### Refinement

**Table:**

Refinement on *F*^2^	Primary atom site location: structure-invariant direct methods
Least-squares matrix: full	Secondary atom site location: difference Fourier map
*R*[*F*^2^ > 2σ(*F*^2^)] = 0.040	Hydrogen site location: inferred from neighbouring sites
*wR*(*F*^2^) = 0.110	H-atom parameters constrained
*S* = 1.04	*w* = 1/[σ^2^(*F*_o_^2^) + (0.0473*P*)^2^ + 1.2513*P*] where *P* = (*F*_o_^2^ + 2*F*_c_^2^)/3
2846 reflections	(Δ/σ)_max_ < 0.001
145 parameters	Δρ_max_ = 0.41 e Å^−^^3^
0 restraints	Δρ_min_ = −0.29 e Å^−^^3^

### Special details

**Table:**

Geometry. All e.s.d.'s (except the e.s.d. in the dihedral angle between two l.s. planes) are estimated using the full covariance matrix. The cell e.s.d.'s are taken into account individually in the estimation of e.s.d.'s in distances, angles and torsion angles; correlations between e.s.d.'s in cell parameters are only used when they are defined by crystal symmetry. An approximate (isotropic) treatment of cell e.s.d.'s is used for estimating e.s.d.'s involving l.s. planes.
Refinement. Refinement of *F*^2^ against ALL reflections. The weighted *R*-factor *wR* and goodness of fit *S* are based on *F*^2^, conventional *R*-factors *R* are based on *F*, with *F* set to zero for negative *F*^2^. The threshold expression of *F*^2^ > σ(*F*^2^) is used only for calculating *R*-factors(gt) *etc*. and is not relevant to the choice of reflections for refinement. *R*-factors based on *F*^2^ are statistically about twice as large as those based on *F*, and *R*- factors based on ALL data will be even larger.

### Fractional atomic coordinates and isotropic or equivalent isotropic displacement parameters (Å^2^)

**Table:**

	*x*	*y*	*z*	*U*_iso_*/*U*_eq_	
Cl1	0.43189 (3)	0.78273 (5)	0.61104 (4)	0.05385 (16)	
Cl2	0.38622 (3)	0.67345 (6)	0.83020 (5)	0.06457 (19)	
N1	0.54978 (8)	0.77484 (13)	0.88700 (13)	0.0370 (3)	
O1	0.60625 (8)	0.77362 (14)	1.06154 (12)	0.0555 (4)	
C11	0.46001 (9)	0.72103 (16)	0.74626 (16)	0.0398 (4)	
H11	0.4904	0.6483	0.7323	0.048*	
C10	0.49952 (9)	0.82044 (15)	0.81777 (15)	0.0372 (4)	
O2	0.48479 (8)	0.93204 (12)	0.81157 (14)	0.0554 (4)	
C9	0.56836 (10)	0.64244 (17)	0.90877 (18)	0.0468 (4)	
H9A	0.5882	0.6034	0.8393	0.056*	
H9B	0.5283	0.5940	0.9347	0.056*	
C5	0.65059 (10)	0.91874 (17)	0.91330 (18)	0.0469 (4)	
C7	0.58735 (10)	0.85716 (17)	0.96905 (16)	0.0431 (4)	
H7	0.5557	0.9221	0.9992	0.052*	
C6	0.67948 (11)	0.8764 (2)	0.8089 (2)	0.0549 (5)	
H6	0.6588	0.8094	0.7696	0.066*	
C8	0.62234 (13)	0.65601 (19)	1.0063 (2)	0.0593 (6)	
H8A	0.6187	0.5872	1.0620	0.071*	
H8B	0.6692	0.6571	0.9741	0.071*	
C1	0.73902 (12)	0.9323 (2)	0.7616 (3)	0.0722 (7)	
H1	0.7579	0.9027	0.6915	0.087*	
C2	0.76916 (14)	1.0306 (3)	0.8190 (3)	0.0880 (10)	
H2	0.8087	1.0684	0.7873	0.106*	
C3	0.74266 (15)	1.0743 (2)	0.9217 (3)	0.0859 (10)	
H3	0.7644	1.1408	0.9602	0.103*	
C4	0.68205 (13)	1.0194 (2)	0.9706 (3)	0.0687 (7)	
H4	0.6635	1.0503	1.0404	0.082*	

### Atomic displacement parameters (Å^2^)

**Table:**

	*U*^11^	*U*^22^	*U*^33^	*U*^12^	*U*^13^	*U*^23^
Cl1	0.0541 (3)	0.0720 (4)	0.0354 (2)	−0.0010 (2)	−0.00397 (19)	0.0028 (2)
Cl2	0.0712 (4)	0.0745 (4)	0.0481 (3)	−0.0336 (3)	0.0014 (2)	0.0033 (2)
N1	0.0418 (7)	0.0300 (7)	0.0392 (8)	0.0004 (5)	−0.0036 (6)	−0.0011 (6)
O1	0.0760 (9)	0.0524 (8)	0.0380 (7)	0.0039 (7)	−0.0131 (7)	0.0019 (6)
C11	0.0440 (9)	0.0380 (9)	0.0374 (9)	0.0004 (7)	−0.0043 (7)	−0.0017 (7)
C10	0.0404 (8)	0.0332 (8)	0.0379 (9)	−0.0010 (7)	−0.0017 (7)	0.0010 (7)
O2	0.0669 (9)	0.0311 (6)	0.0684 (9)	0.0031 (6)	−0.0216 (7)	0.0007 (6)
C9	0.0557 (11)	0.0333 (9)	0.0513 (11)	0.0084 (8)	−0.0050 (9)	−0.0002 (7)
C5	0.0479 (10)	0.0323 (8)	0.0604 (12)	0.0007 (7)	−0.0221 (9)	0.0043 (8)
C7	0.0518 (10)	0.0380 (9)	0.0394 (9)	0.0051 (8)	−0.0115 (8)	−0.0054 (7)
C6	0.0491 (10)	0.0543 (12)	0.0614 (13)	−0.0079 (9)	−0.0096 (9)	0.0089 (10)
C8	0.0771 (14)	0.0455 (11)	0.0552 (12)	0.0101 (10)	−0.0184 (11)	0.0056 (9)
C1	0.0502 (12)	0.0794 (16)	0.0871 (18)	−0.0083 (12)	−0.0058 (12)	0.0259 (14)
C2	0.0569 (14)	0.0697 (17)	0.138 (3)	−0.0160 (13)	−0.0268 (17)	0.0369 (19)
C3	0.0719 (16)	0.0409 (11)	0.145 (3)	−0.0149 (11)	−0.0536 (19)	0.0079 (15)
C4	0.0716 (14)	0.0397 (10)	0.0948 (18)	0.0036 (10)	−0.0389 (14)	−0.0066 (11)

### Geometric parameters (Å, °)

**Table:**

Cl1—C11	1.7563 (18)	C5—C4	1.389 (3)
Cl2—C11	1.7803 (19)	C5—C7	1.517 (3)
N1—C10	1.335 (2)	C7—H7	0.9800
N1—C7	1.468 (2)	C6—C1	1.395 (3)
N1—C9	1.471 (2)	C6—H6	0.9300
O1—C7	1.423 (2)	C8—H8A	0.9700
O1—C8	1.431 (3)	C8—H8B	0.9700
C11—C10	1.533 (2)	C1—C2	1.360 (4)
C11—H11	0.9800	C1—H1	0.9300
C10—O2	1.220 (2)	C2—C3	1.355 (5)
C9—C8	1.524 (3)	C2—H2	0.9300
C9—H9A	0.9700	C3—C4	1.414 (4)
C9—H9B	0.9700	C3—H3	0.9300
C5—C6	1.385 (3)	C4—H4	0.9300
			
C10—N1—C7	120.89 (14)	O1—C7—H7	109.7
C10—N1—C9	128.35 (15)	N1—C7—H7	109.7
C7—N1—C9	110.07 (14)	C5—C7—H7	109.7
C7—O1—C8	105.92 (14)	C5—C6—C1	121.4 (2)
C10—C11—Cl1	111.07 (12)	C5—C6—H6	119.3
C10—C11—Cl2	107.69 (12)	C1—C6—H6	119.3
Cl1—C11—Cl2	109.34 (10)	O1—C8—C9	104.80 (16)
C10—C11—H11	109.6	O1—C8—H8A	110.8
Cl1—C11—H11	109.6	C9—C8—H8A	110.8
Cl2—C11—H11	109.6	O1—C8—H8B	110.8
O2—C10—N1	123.59 (16)	C9—C8—H8B	110.8
O2—C10—C11	121.57 (16)	H8A—C8—H8B	108.9
N1—C10—C11	114.83 (14)	C2—C1—C6	119.2 (3)
N1—C9—C8	101.35 (15)	C2—C1—H1	120.4
N1—C9—H9A	111.5	C6—C1—H1	120.4
C8—C9—H9A	111.5	C3—C2—C1	121.2 (3)
N1—C9—H9B	111.5	C3—C2—H2	119.4
C8—C9—H9B	111.5	C1—C2—H2	119.4
H9A—C9—H9B	109.3	C2—C3—C4	120.4 (2)
C6—C5—C4	118.5 (2)	C2—C3—H3	119.8
C6—C5—C7	122.59 (17)	C4—C3—H3	119.8
C4—C5—C7	118.9 (2)	C5—C4—C3	119.3 (3)
O1—C7—N1	102.94 (14)	C5—C4—H4	120.3
O1—C7—C5	111.96 (15)	C3—C4—H4	120.3
N1—C7—C5	112.55 (15)		

### Hydrogen-bond geometry (Å, °)

**Table:**

*D*—H···*A*	*D*—H	H···*A*	*D*···*A*	*D*—H···*A*
C11—H11···O2^i^	0.98	2.40	3.312 (2)	156

Symmetry codes: (i) −*x*+1, *y*−1/2, −*z*+3/2.

## References

[bb1] Agami, C. & Couty, F. (2004). *Eur. J. Org. Chem.* **69**, 677–685.

[bb2] Bruker (1998). *SMART* and *SAINT* Bruker AXS Inc., Madison, Wisconsin, USA.

[bb3] Daniele, D. B., Luciano, S. & Luca, E. (2007). *Phytochemistry*, **68**, 2614–2618.

[bb4] Fu, Y., Fu, H. G., Ye, F., Mao, J. D. & Wen, X. T. (2009). *Synth. Commun.* **39**, 2454–2463.

[bb5] Guirado, A., Andreu, R. & Galvez, J. (2003). *Tetrahedron Lett.* **44**, 3809–3841.

[bb6] Hatzios, K. K. (2004). *Weed Sci.* **52**, 454–467.

[bb7] Sheldrick, G. M. (1996). *SADABS* University of Göttingen, Germany.

[bb8] Sheldrick, G. M. (2008). *Acta Cryst.* A**64**, 112–122. 10.1107/S0108767307043930 18156677

[bb9] Tararov, V. I., Kadyrov, R., Monsees, A., Riermeier, T. H. & Boerner, A. (2003). *Adv. Synth. Catal.* **345**, 239–245.

